# Validation of an Electronic Visual Analog Scale mHealth Tool for Acute Pain Assessment: Prospective Cross-Sectional Study

**DOI:** 10.2196/13468

**Published:** 2020-02-12

**Authors:** Carles Escalona-Marfil, Andrea Coda, Jorge Ruiz-Moreno, Lluís Miquel Riu-Gispert, Xavier Gironès

**Affiliations:** 1 Facultat de Ciències de la Salut de Manresa Universitat de Vic–Universitat Central de Catalunya Manresa Spain; 2 University School of Health and Sport University of Girona Salt (Girona) Spain; 3 School of Health Sciences, Faculty of Health and Medicine Priority Research Centre for Health Behaviour, Hunter Medical Research Institute The University of Newcastle Callaghan Australia; 4 MIXESTAT SL Barcelona Spain

**Keywords:** pain, visual analog pain scale, pain measurement, mobile phone, mHealth, validation, tablet

## Abstract

**Background:**

Accurate measurement of pain is required to improve its management and in research. The visual analog scale (VAS) on paper format has been shown to be an accurate, valid, reliable, and reproducible way to measure pain intensity. However, some limitations should be considered, some of which can be implemented with the introduction of an electronic VAS version, suitable to be used both in a tablet and a smartphone.

**Objective:**

This study aimed to validate a new method of recording pain level by comparing the traditional paper VAS with the pain level module on the newly designed Interactive Clinics app.

**Methods:**

A prospective observational cross-sectional study was designed. The sample consisted of 102 participants aged 18 to 65 years. A Force Dial FDK 20 algometer (Wagner Instruments) was employed to induce mild pressure symptoms on the participants’ thumbs. Pain was measured using a paper VAS (10 cm line) and the app.

**Results:**

Intermethod reliability estimated by ICC(3,1) was 0.86 with a 95% confidence interval of 0.81 to 0.90, indicating good reliability. Intramethod reliability estimated by ICC_a_(3,1) was 0.86 with a 95% confidence interval of 0.81 to 0.90, also indicating good reliability. Bland-Altman analysis showed a difference of 0.175 (0.49), and limits of agreement ranged from –0.79 to 1.14.

**Conclusions:**

The pain level module on the app is highly reliable and interchangeable with the paper VAS version. This tool could potentially help clinicians and researchers precisely assess pain in a simple, economic way with the use of a ubiquitous technology.

## Introduction

The ability to record pain level objectively represents a crucial aspect for allied health professionals in monitoring the effectiveness of the prescribed interventions. Clinicians may experience difficulties in conducting frequent assessments; therefore, different primary outcomes, such as recording pain progression, may rely entirely on recall during appointments [1].

The traditional visual analog scale (VAS) on paper format has been shown to be accurate, valid, reliable, and reproducible [2]. However, despite the widespread use of the paper VAS version, limitations should be considered such as the need for the allied health professional to measure the pain data using a ruler and manually transcribing its values into electronics notes and participant noncompliance to paper diaries in clinical trial [3]. Pain data acquired may be subject to potential transcription error, typing mistakes, and potential backfilling entries in paper pain diaries [4].

Growing evidence exists in new interactive methods in recording pain level using real-time data capture technology with multidimensional electronic pain diaries (e-Ouch), which has been validated in adolescents diagnosed with juvenile idiopathic arthritis [5]. In addition, a recent accuracy, validity, and reliability trial strongly suggested that iPadVAS provides a user-friendly and efficient method to collect pain levels in healthy older adults [6]. The iPasVAS settings impede participants from scoring a line outside the VAS line, preventing invalid data from being recorded from clinicians [6]. These instruments can be designated as electronic VAS (eVAS).

The validity and reliability of the apps used to monitor pain progression require further research prior to be introduced into everyday clinical settings. Different devices have been already introduced to compare the paper VAS as a gold standard.

The cost of this smart technology is a critical factor that may limit its introduction into different clinical and research settings. However, the cost for these user-friendly smart devices is gradually becoming more affordable, and they are increasingly present in the market worldwide [7]. Globally, the number of people subscribed to mobile services is 5.1 billion (67% of the global population), with an average annual growth rate of about 5% [8]. Also to be noted is that in the next 7 years, about 710 billion people will subscribe to mobile services for the first time [8]. Finally, the introduction of these more affordable smart devices in different aspects of pain management may improve the engagement and understanding of symptom progression, drug adherence, and overall clinical outcomes.

This prospective observational cross-sectional study aims to explore new methods of recording pain level in health adults by comparing the traditional paper VAS with an eVAS from the pain level module included in the Interactive Clinics app (Bit Genoma Digital Solutions SL).

## Methods

### Design, Population, and Sample

A prospective observational cross-sectional study was designed, and students and staff aged 18 to 65 years from the University of Manresa in the University of Vic–Central University of Catalonia (UVic-UCC) were invited to take part to this project. Inclusion criteria consisted of participants who were not currently taking medications that could have compromised the perception or sensation of pain. Participants were excluded if they suffered from finger nail pathologies or inability to fully understand the pain scale due to language or mental health issues.

### Measuring Instruments

**Figure 1:**
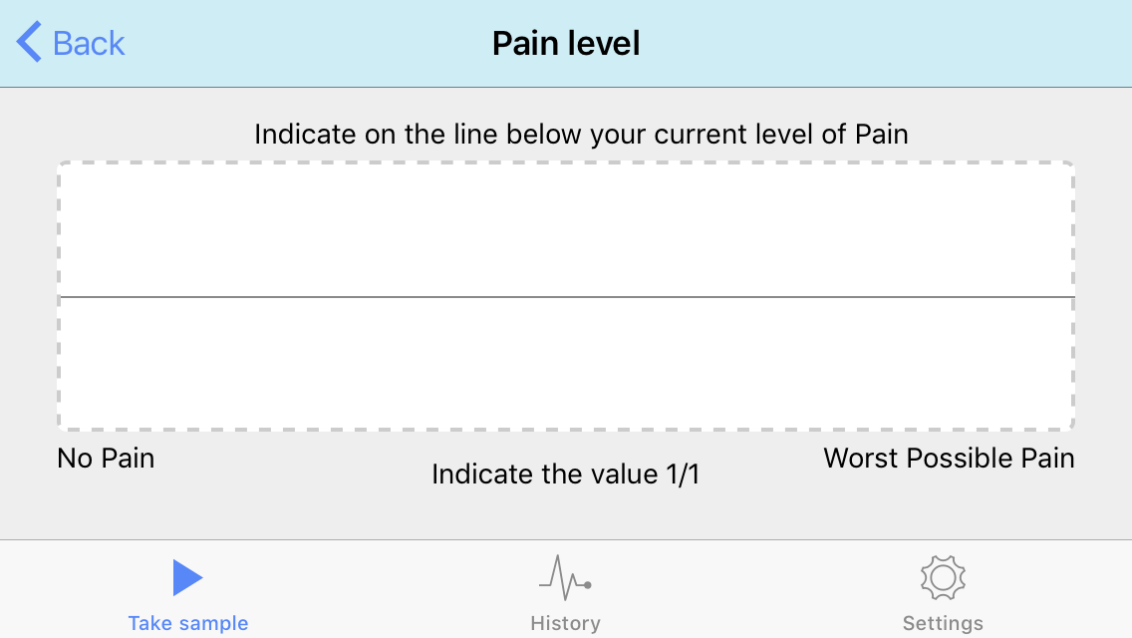
Screenshot of the pain level module on the Interactive Clinics app.

**Figure 2:**
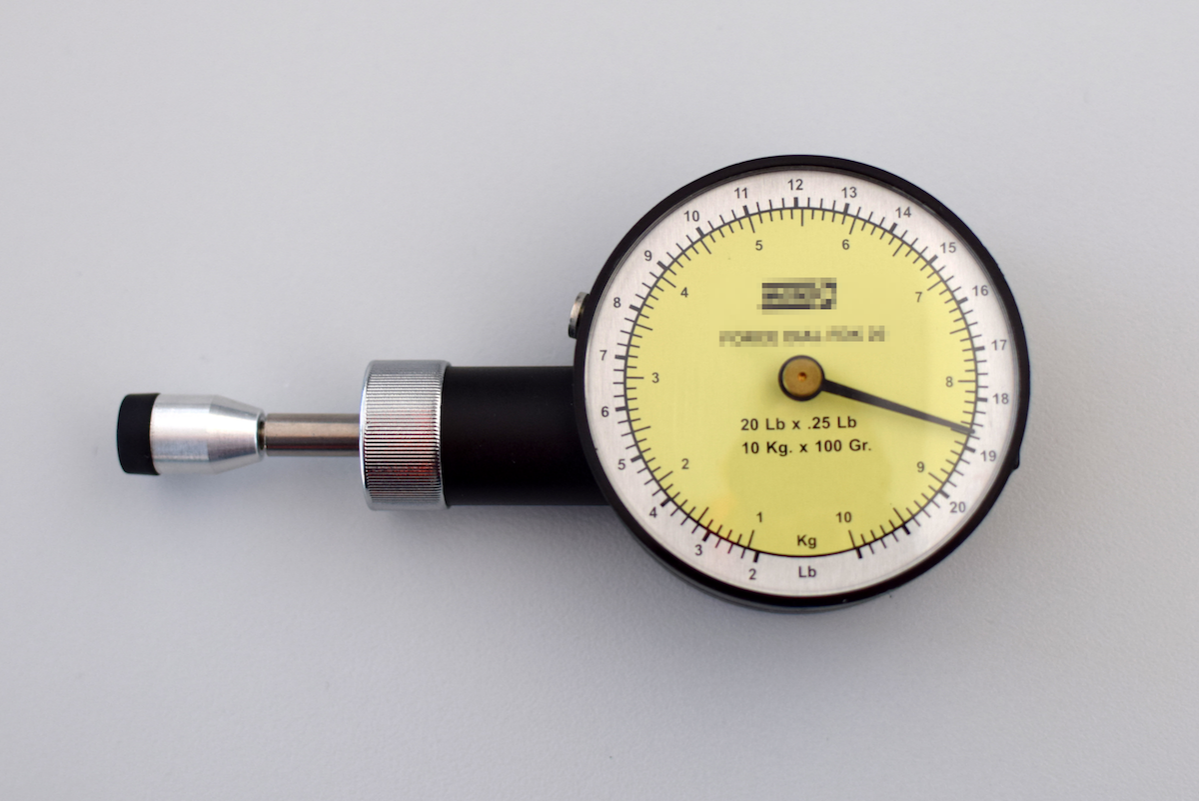
Force Dial FDK 20 algometer (Wagner Instruments) used to cause acute pain.

To measure pain, a VAS was used on paper and on an electronic tablet. For data collection on paper, a 12×7.5 cm sheet with a 10 cm horizontal line and two 6 cm vertical lines drawn at its edges was used. Electronic measurements were made using the pain level module included in the Interactive Clinics app installed on a 7-inch Galaxy Tab 3 CE0168 with Android operating system (Samsung), which displays a plain gray line on white background (Figure 1). To cause the local pain, a Force Dial FDK 20 algometer (Wagner Instruments) with a rubber 1 cm^2^ circular end was used (Figure 2).

### Protocol to Perform Validation of Electronic Visual Analog Scale

Prior to the procedure, participants were assessed through a short interview to check if they fulfilled the selection criteria, and they were asked about their personal data. One researcher explained the procedure and after reading the information sheet, participants signed the informed consent.

**Figure 3:**
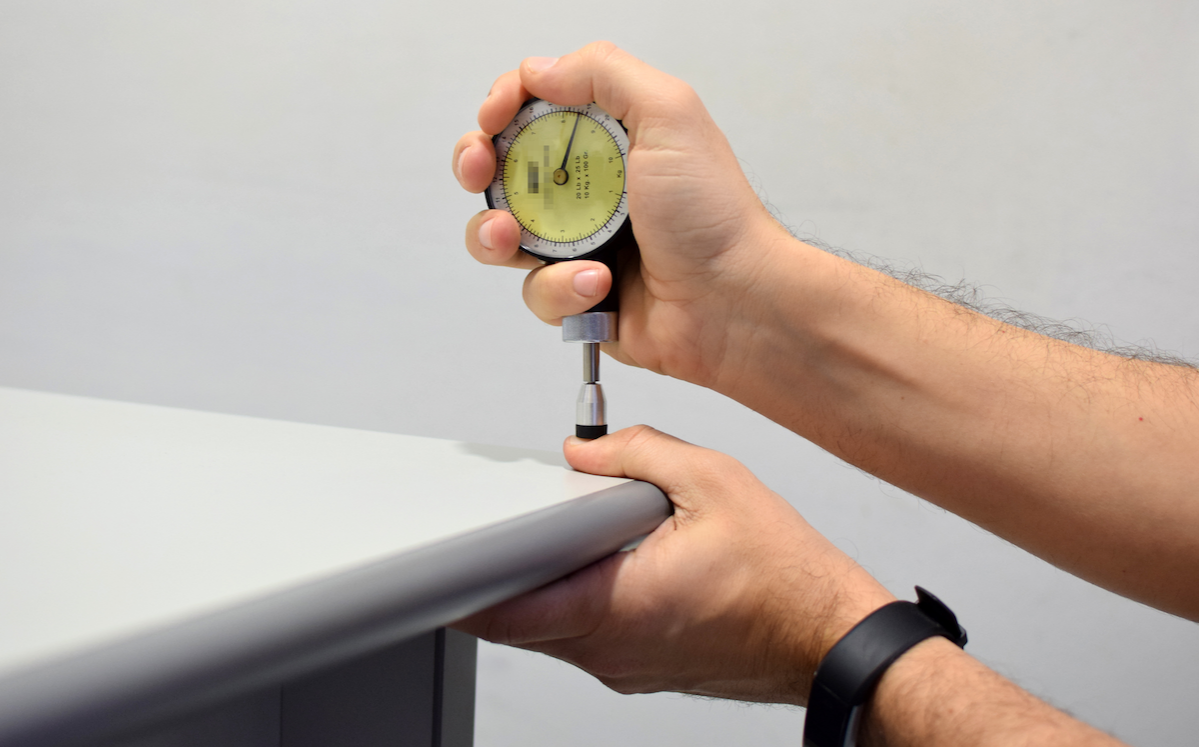
Pressure application procedure with the algometer.

Participants were sitting on a chair in front of a rigid wooden table with the thumb on the table and the other fingers under it. The pulp of the thumb was touching the table and the nail looking up. Since pain is an alarm sign, it appears much earlier that tissue damage. Taking that into account to overcome the pain threshold and ensure that a certain pain was caused, a vertical 8.5 kg force was applied with the 1 cm^2^ rubber end of the Force Dial for 3 seconds on the thumb at the midpoint of the nail, over the lunule but not pressing the eponychium (Figure 3).

After the end of the pressure, participants were asked to record their pain drawing a short vertical line on the horizontal line of the paper, considering that the left end corresponded to no pain and the right end to the worst pain imaginable. Afterward, they were asked to record their pain on the app, pointing with one finger on the horizontal line of the tablet screen, with the tablet in horizontal position (landscape) so the line was longer and easier to manipulate (Multimedia Appendix 1).

To increase reliability, the procedure (pressure, paper, tablet) was repeated twice, with a minimum period of 5 minutes between attempts. The authors decided not to randomize the first tool to register pain in order to create a more standardized and repeatable protocol that could even be easily introduced in a clinical situation; furthermore, the possible sequence effect was previously verified by means of a panel data regression in a random sample of similar sequenced individuals, not observing such effect.

So as not to create a bias on the patient pointing, any previous recording was removed. Participants could not see the paper while they were pointing to the tablet or the next paper, and they were not informed of their results until they had finished the procedure.

Results on paper were measured using a 12 cm plastic ruler. The app showed the results on the screen (after pressing a button, so the participants could not see their results) and were directly recorded in an electronic form.

### Statistical Analysis

Summary statistics for eVAS were calculated by splitting measurement and method. Two approaches have been used to evaluate agreement of the two methods: intraclass correlation coefficient (ICC) analysis and exploratory Bland-Altman plot analysis. SAS 9.4 (SAS Institute Inc) and Stata 15 (StataCorp LLC) were used for statistical analysis.

### Intermethod and Intramethod Agreement Analysis

A mixed factorial model was employed to derive two ICCs according to Shrout-Fleiss reliability fixed set: one coefficient as a measure of intermethod reliability, ρ, estimated by ICC(3,1). This coefficient is defined as the correlation between VAS values from different methods in the same subject and same replication. The other intraclass coefficient, γ, estimated by ICC_a_(3,1), was used as a measure of intramethod reliability. This is defined as the correlation between VAS values in the same method and same subject. A 2-way balanced mixed analysis of variance model without interaction, random subject effect, and fixed method effect were fitted in order to estimate ICCs. The mean of squares for subjects, subject-method interaction, and errors from components of variance were also calculated. Statistical inference of the ICCs was performed with confidence intervals and test of hypothesis [9]. In order to improve reliability coefficients, a 95% confidence interval was calculated from the estimated sum of squares. The research hypotheses for both ICCs were that ρ and γ exceed the value of .80. In order to specify the precision of the estimated ICC, the length of the 95% confidence interval was expressed as a function of the ICC value. Given that it was not possible to increase the number of methods to evaluate VAS, the number of subjects was increased. With 204 ratings per method (102 subjects with 2 replicates per subject) and an anticipated value of ICC of at least .80, an acceptable length for the 95% confidence interval will be less than or equal to 0.08. Good agreement among methods was evaluated plotting both methods against subject and performing a Wilcoxon rank-sum test.

### Bland-Altman Analysis

The considered difference was eVAS measurement minus paper measurement. This graphical approach displays the differences between methods as measure of imprecision against the mean value of measures as measure of magnitude [10]. In the present Bland-Altman analysis, each subject is measured by each method twice, and it is assumed that the overall response mean varies during the data gathering period. In order to perform the analysis, limits of agreement was carried out and defined as mean of differences ±1.96*SD_diff_. This standard deviation is the square root of the variance as a sum of variance for repeated differences between the two methods on the same subject and variance for differences between the average of the two methods across subjects. Then, a 1-way analysis of variance was fitted with the differences as response to obtain both variances. Assumptions of the model, constant within subject variance, assumption of independence between repeated differences inside a subject, and random or systematic variation were assessed in a graphical approach. Normal distribution of the differences was verified using Kolmogorov-Smirnov or Shapiro-Wilk tests, displays of histogram, and quantile-quantile plot. Confidence interval estimation for limits of agreement (LoA) were computed using both Delta and method of variance estimates recovery methods. As the second seems more accurate in small-to-moderate sample sizes, it was presented in this paper. An SAS macro implementing calculations for confidence intervals for LoA with multiple measurements per individual was applied [11].

### Ethics

Written informed consent was obtained from each participant before data collection stating (1) they understood they would experience moderate pain, (2) the experimental procedures were clearly explained, and (3) they could withdraw at any time without prejudice. This study was approved by the UVic-UCC research ethics committee in Vic (Barcelona).

## Results

### Intermethod and Intramethod Agreement Analysis

**Figure 4:**
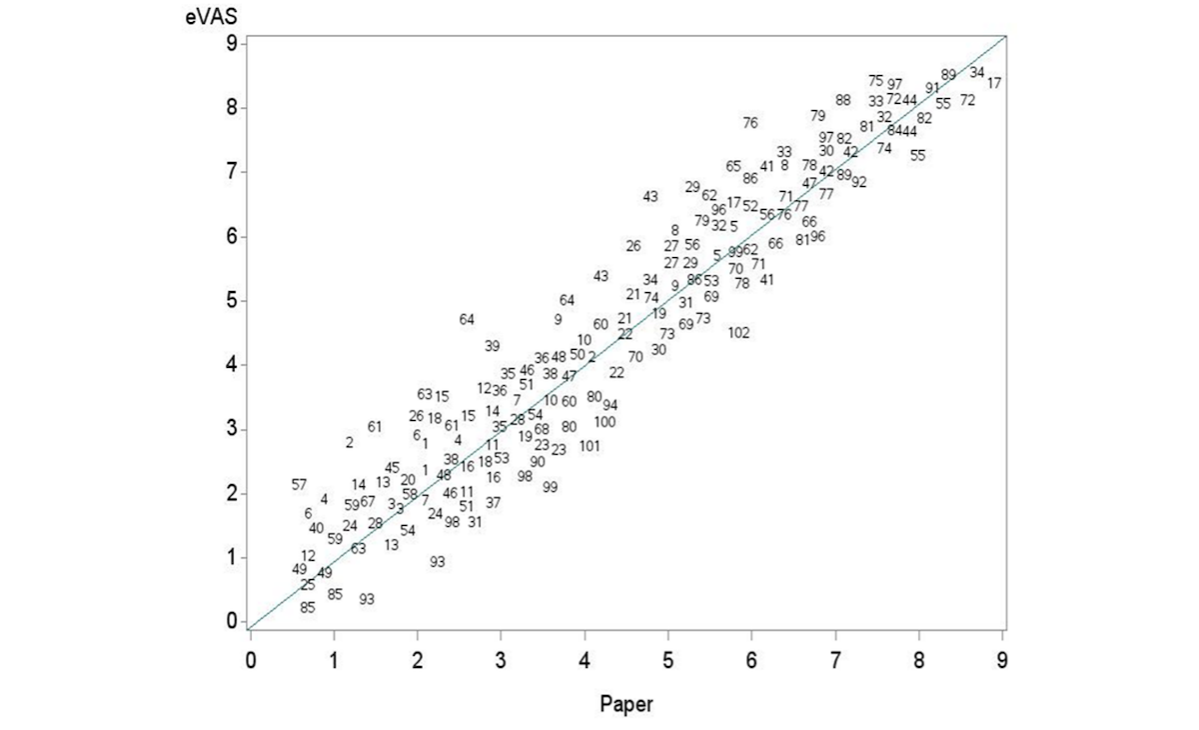
Scatter plot of the data (points are represented by subject number).

**Figure 5:**
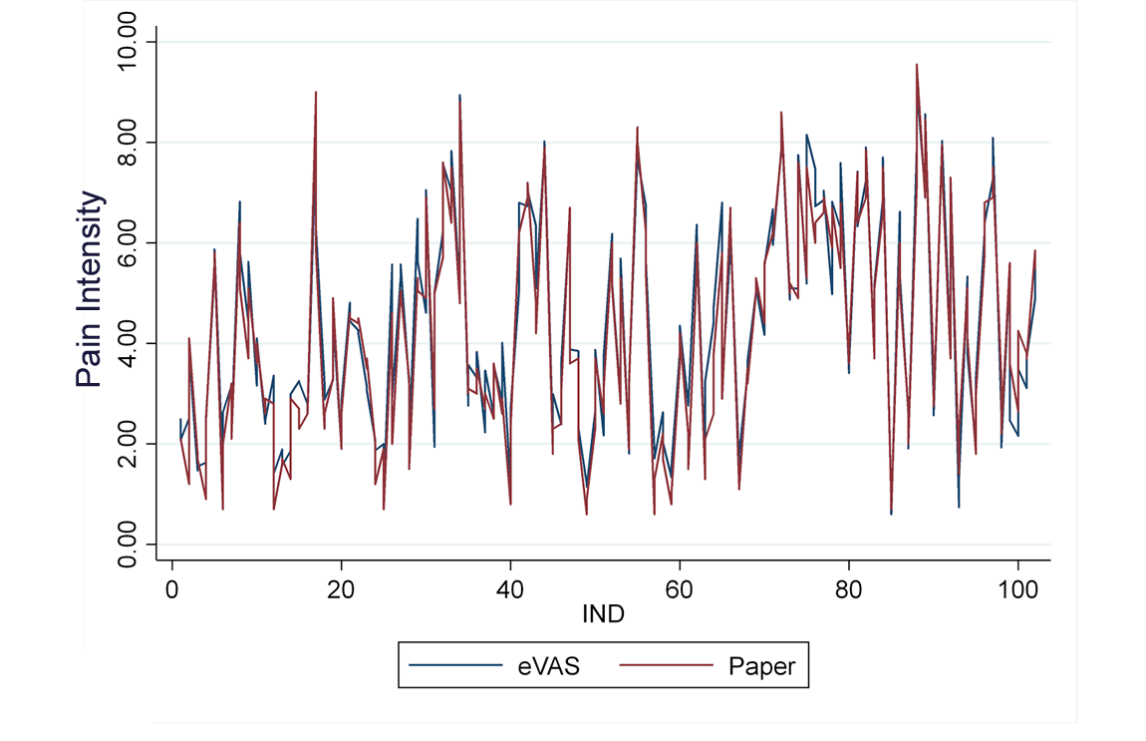
Rating data for the two methods.

Table 1 shows summary statistics for VAS measurements by measurement order and instrument (eVAS and paper). Differences between methods of median values are 0.13 and 0.10 for first and second measurements, respectively. In Figure 4, the scatter plot for eVAS versus paper for every subject (numbered) is displayed, showing a good agreement between the two methods. Figure 5 shows a good agreement indicating no difference between eVAS and paper VAS measurements and suitability in using ICC mixed factorial design. The 2-sample Wilcoxon rank-sum test for comparing methods was not significant (*P*=.41). The intermethod reliability estimated by ICC(3,1) reached the value of .86 with a 95% confidence interval of 0.81 to 0.90 indicating good reliability. The intramethod reliability estimated by ICC_a_(3,1) reached the value of .86 with a 95% confidence interval of 0.81 to 0.90, also indicating good reliability [12]. For both coefficients, the length of the interval was 0.08. Our data supports the research hypotheses stating ρ >0.8 (*P*=.006) and γ >0.8 (*P*=.01).

**Table 1:** Summary statistics for visual analog scale measurements (N=102).

Attempt and instrument		Visual analog scale	
		Mean (SD)	Median (min, max)
**1**			
	eVAS^a^	4.20 (2.09)	3.78 (0.74, 8.11)
	Paper	4.04 (2.10)	3.65 (0.60, 9.00)
**2**			
	eVAS	4.52 (2.19)	3.95 (0.60, 9.15)
	Paper	4.33 (2.23)	4.05 (0.70, 9.55)

^a^eVAS: electronic visual analog scale.

### Bland-Altman Analysis

**Figure 6:**
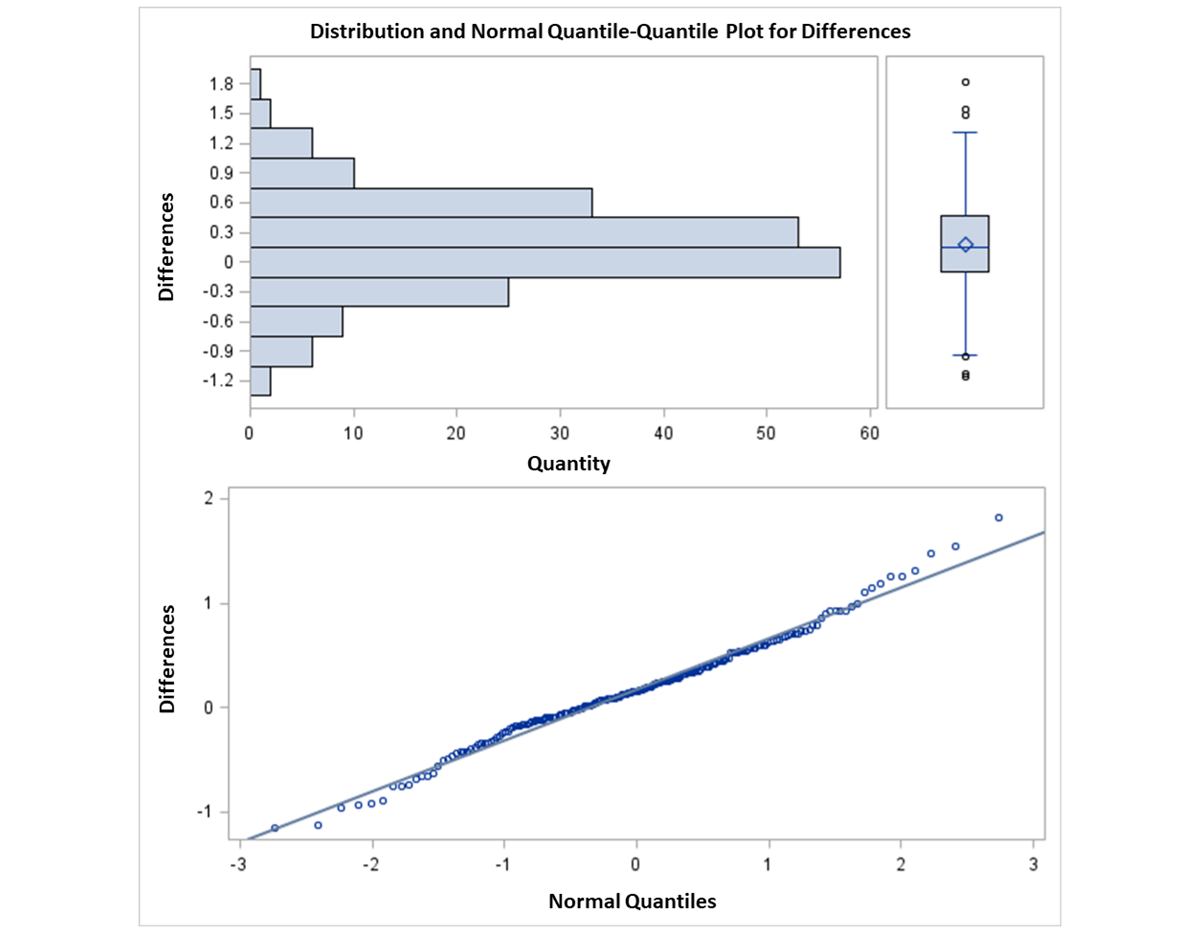
Normal distribution of the differences.

Normal distribution of the differences was checked by means, Kolmogorov-Smirnov test (*P*=.10), Shapiro-Wilk test (*P*=.09), and histogram and quantile-quantile plots (Figure 6).

**Figure 7:**
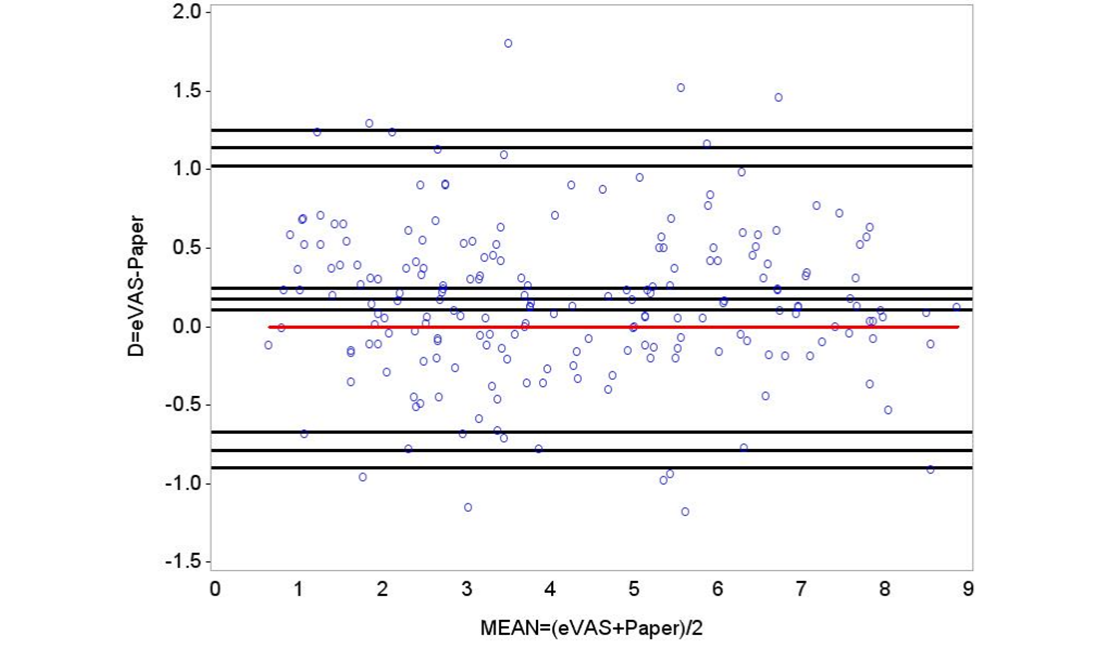
Bland-Altman plot of differences between methods against the average of the two. Red line is the zero reference value for difference. Black lines represent the sample mean, limits of agreement, and 95% confidence intervals.

The Bland-Altman plot is displayed in Figure 7. The lines show limits of confidence for the mean and LoA, and the red line shows the zero-reference value for the differences. The red line is the zero-line used to assess the discrepancy of the observed mean difference. The Bland-Altman plot method only defines the intervals of agreements; whether those limits are acceptable will depend on the investigator. An acceptable range must be previously established, based on clinical or biological considerations or other goals [13]. The limit of 1.30 is considered a clinically significant difference between the two methods [14]. The mean of the differences was 0.175 (SD 0.49), meaning there exists a bias of 0.175 units (Figure 7). The confidence interval for the mean of differences ranges from 0.10 to 0.24, not covering the value of 1.30. The LoA range from –0.79 to 1.14, appearing to fit the data well; they represent the range of values inside which 95% of differences are expected between eVAS and paper assuming a normal distribution. Results measured with eVAS may be 0.79 units below or 1.14 units above VAS paper results (Figure 7). The precision of LoA was computed by means of 95% confidence intervals. Lower LoA limits ranged from –0.90 to –0.67, and upper LoA ranged from 1.02 to 1.25; these figures indicating the magnitude of the systematic difference. Considering lower limit of lower LoA and upper limit of upper LoA, it is possible to observe the value of ±1.30 is not covered.

## Discussion

### Principal Findings

A mild pain was caused with an algometer in the thumbnail in two attempts and measured on paper (4.04 [SD 2.10] and 4.33 [SD 2.23]) and electronic (4.20 [SD 2.09] and 4.52 [SD 2.19]) VASs. Good intermethod (ICC[1,3]=.86) and intramethod (ICC_a_[1,3]=.86) reliability was supported. Bland-Altman analysis showed a difference of 0.18 (SD 0.49), and LoA ranged from –0.79 to 1.14.

The introduction of mobile devices and tablets in everyday health application is becoming increasingly common [15-17]. New smart health technologies are now available for clinicians and researchers, which may positively impact patient compliance to prescribed treatment and overall health care [18]. The use of mobile apps in pain management has been demonstrated to have a number of benefits, especially in clinical settings: pain apps are easy to use and usually welcomed by patients and clinicians [19,20]. Some concerns may arise in introducing mobile health (mHealth) in an elderly population (aged 65 years and older); however, there is growing evidence of accessibility and successful use of mobile pain apps in this population [21]. It is well recognized that pain assessment is the initial step in the early identification of many pathologies, and it is frequently adopted in effective clinical management plans [22].

However, the quality of some apps is still questionable, especially for pain management [23]. In our study, interrater agreement and an exploratory Bland-Altman plot analysis were presented in order to reach agreement between methods. Regarding ICC analysis, mean of squares from intrasubject and subject-method interaction were very small (0.65) compared with mean of squares from subject (16.46). No systematic effect in methods was found, even when inducing high values of ICC.

Bland-Altman analysis reported no interaction to subject by method or correlation between differences. The analyses of data replicates was accounted for, instead of the mean values, enabling the comparison of repeatability of methods and obtaining more realistic LoA when considering both within and between subject difference variation. Averaging the subject replications would remove variation within the subject. The calculated LoA would be narrower, especially if both within and between subject variations were similar.

Compared with the traditional paper version of the VAS as a gold standard, the results of this study provide very strong evidence of the validity and reliability of the electronic version of the pain level module on the Interactive Clinics app when assessing acute pain in adults. The mean of pain registered by the subjects only differed by 0.18 units between paper and eVAS, a very small difference compared with 1.30 units considered clinically significant [13]. From the obtained LoA, results measured with eVAS may be 0.79 units below or 1.14 units above paper results (Figure 5). It was also possible to estimate the precision of the LoA as 95% confidence intervals. Considering a 95% confidence interval on lower limit of lower LoA and a 95% confidence interval on upper limit of upper LoA, the clinically significant difference of ±1.30 is still not covered.

One of the most widely adopted instruments to measure pain level is the VAS, which has previously proven its validity and reliability as a pain categories tool [24-26]. Pain is a subjective experience, and therefore it may be difficult to measure in terms of physiologic response unless using complex and expensive materials [27]. Hence, patient’s self-reported measures are valuable and frequently used in clinical and research settings.

In order to compare the assessment of pain between the paper version and the electronic device, acute pain was caused to each subject by means of an algometer. This method has been previously considered easy to operate and reliable [28,29]. Furthermore, it has been validated to determine pain threshold [30,31], and it has been found repeatable and stable [32]. As expected, despite the exact same stimulus of pressure being applied to each subject, individual perception was recorded to be different.

The paper VAS format presents with some limitations, especially when measuring the evolution of pain in noninstitutionalized patients. There are some alternatives. The numeric rating scale (NRS) and the verbal rating scale (VRS) can be performed by phone and have demonstrated different levels of consistency and validity. The VAS showed the highest scores [33,34]. Bijur et al [35] concluded that NRS was strongly correlated with VAS in emergency patients, making NRS suitable for these patients. However, VRS and VAS are not interchangeable when measuring pain, whether chronic pain [36] or chronic/idiopathic, nociceptive, and neuropathic pain [37]. As a consequence, the measurement instrument used before, during, and after a surgical procedure should be the same.

Compared with the VRS and NRS, the eVAS is self-reported and self-administered, which allows an unlimited number of measures regarding research costs from an economic and time perspective. This is a significant advantage, especially considering the increasing number of noninstitutionalized postoperative and chronic pain patients. The electronic devices facilitate documentation management and may encourage active patient participation [6]. Furthermore, the eVAS, in an adequate app framework, automatically enables a precise record of the day and time the assessments have been performed, reducing potential human error and time for data collection. Consequently, pain level can be assessed at different time intervals during the day and as frequently as desired. With increased awareness of a patient’s progression of pain intensity, clinicians may be capable of providing more accurate analgesic strategies and improved clinical management. For example, medication administration can be tailored to prevent symptoms during specific times of the day by increasing its power (dosage or active principle) accordingly.

A recent systematic literature review reported that no comparisons had been made between the VAS in paper-and-pen versus electronic versions for pain assessment [38]. However, previous comparisons have been made regarding appetite. The Apple Newton electronic appetite rating system was determined to be as sensitive and reliable as the paper method [39]. Other studies support the use of electronic versions of the VAS for appetite assessment; however, although no superiority was found in terms of validity, it was highlighted that data are not interchangeable between electronic and paper versions [40-42]. Another study compared eVAS, eNRS, and the electronic version of the Roland-Morris Disability Questionnaire in patients with low back pain [43] and concluded they were comparable with their paper versions.

One main difference reported between the studied app and other previously used devices is the actions that the subjects must perform to confer their results. While in most of the electronic linear scales subjects must place their finger on one end of the line (usually on the left side, corresponding to zero) and slide it until the desired point on the line, in this app subjects simply cast their mark directly on the line, replicating more closely the motion used with the traditional paper VAS. This new feature may provide a higher reliability between devices.

### Limitations

Some limitations should be outlined as part of this study. Although no sequence effect (paper or electronic first) has been demonstrated through an ad hoc previous analysis, future papers may take into account its randomization.

A practical question for future research is whether a single patient using the same device will simply trace the fingerprint left on the screen, especially during successive and repeated recordings. A feasible solution to prevent the patient tracing the previous fingerprint left on the screen is to simply ensure that the subject or data collector cleans the screen after each recording. Regarding our study, it must be noted that all of the electronic measures were made using one single device, a tablet with a 7-inch screen; in order to increase validity, future studies should adopt other tablet screen sizes and include smartphones. Another limitation of the study is that acute pain was initiated to record the desired outcome measured. In order to fully investigate digital symptom progression, future studies may include other categories of pain.

### Conclusions

The eVAS on the Interactive Clinics app has been demonstrated to be highly reliable and consistent with paper version results. Fully understanding the impact that pain progression has on individual patients has long been a challenge for clinicians. The introduction of this reliable, safe, and noninvasive mHealth solution may have the potential to achieve enduring changes in improving patient awareness of their progression of pain.

Future research is needed to further explore the feasibility of the app using other tablet screen sizes and smartphones accessible by the wider population. Finally, the introduction of this novel translated research approach may significantly increase the quality of reliable data accessible to clinicians to address pain-related issues.

## Appendix

Multimedia Appendix 1 The protocol used to cause mild pain and to assess it with paper and electronic Visual Analogue Scales.

## Abbreviations

e-OUCH: electronic pain diary
eVAS: electronic visual analog scale
ICC: intraclass corellation coefficient
LoA: limits of agreement
mHealth: mobile health
NRS: numeric rating scale
UVic-UCC: University of Vic–Central University of Catalonia
VAS: visual analog scale
VRS: verbal rating scale
